# The experience of nurses when providing care across acts that may be perceived as death hastening: A qualitative evidence synthesis

**DOI:** 10.1177/02692163251331162

**Published:** 2025-04-29

**Authors:** Victoria Ali, Nancy Preston, Laura Machin, Jackie Malone

**Affiliations:** 1 Lancaster University, Lancaster, UK; 2 Bradford Teaching Hospitals NHS Foundation Trust, Bradford, UK; 3 Imperial College London, London, UK; 4 University of Huddersfield, Huddersfield, UK

**Keywords:** Assisted suicide, nurse, qualitative research, palliative care, withholding treatment, terminal care, experience, palliative sedation, voluntary stopping eating and drinking, assisted dying

## Abstract

**Background:**

Nurses can be involved in interventions that they perceive as hastening death. These interventions may intentionally cause death, as in the case of assisted dying or result in death as an unintended consequence, such as when life-sustaining treatment is withdrawn. There is increasing evidence regarding nurses’ experiences of providing care in these separate contexts. However, it remains less clear whether parallels exist in experiences across various acts that nurses might consider death hastening.

**Aim:**

To synthesise qualitative research findings on the lived experiences of nurses when involved with acts that may be perceived as death hastening.

**Design:**

A qualitative evidence synthesis utilising thematic synthesis.

**Data sources:**

An initial search of CINHAL, PsychInfo and Medline was undertaken in December 2022 and updated in August 2024. Papers were quality assessed using the Joanna Briggs Institute Critical Appraisal Checklist for Qualitative Research.

**Results:**

Twenty-three papers were included in the review. An overarching theme linked to the emotional labour required to provide care was developed. Three sub-themes influence emotional labour: (1) experiencing personal and professional conflicts, (2) the provision of ‘normal(ised)’ care and (3) perceptions of palliative care as a proxy for hastening death.

**Conclusions:**

This synthesis demonstrates that nurses experience significant emotional labour across acts that may be perceived as death hastening. The level of emotional labour is influenced by nurses’ uncertainty of the ethical and moral status of these interventions and navigating these uncertainties alongside colleagues, patients and those important to them during care delivery.

**What is already known about the topic?**

- Nurses deliver care for patients and those important to them across acts that may intentionally or potentially hasten death, navigating this care within the boundaries of healthcare systems and professional regulation.
- The increase in permissive legislation relating to assisted dying is challenging healthcare professionals to consider how an assisted death sits alongside accepted or ‘traditional’ healthcare practices at the end of life.
- Providing care in these situations can be challenging and requires emotional labour to navigate.

**What this paper adds?**

- This review allows recognition of how the emotional labour involved in providing care, and its subsequent impact, is often better recognised within assisted dying than for other acts that may be perceived as death hastening.
- The ‘normalising’ of care, and consequently dying, within acts that may be perceived as hastening death limits the recognition of the emotional labour required for nurses to provide care in these circumstances.
- When supporting a patient through an assisted death, nurses focus on optimising the experience for the patient, whereas in other acts that may hasten death, nurses’ primary focus is on the experience of those present with the patient.

**Implications for practice, theory or policy**

- The impact on nurses’ emotional well-being due to the expectation to engage in significant emotional labour, in all care that may be perceived as death hastening, should be considered in daily practice, policy and organisational structure.
- The provision of emotional support should be considered for nurses when involved in the delivery of care that may hasten death, either through intentional acts (an assisted death) or unintended consequence of the care.
- Normalising care that may be perceived as death-hastening can impact nurses’ feelings of agency within care delivery and may need to be considered in jurisdictions with permissive assisted dying legislation as these practices embed within organisations.

## Background

An increasing number of countries permit assisted dying, an act that intentionally hastens a patient’s death. Other acts within healthcare have the potential to hasten death, although are not used with this intention. Examples of such care might be the use of sedation or the withdrawal of life-sustaining treatment.^1,2^ On a clinical, conceptual, and philosophical level, what encompasses ‘hastened death’ remains poorly defined with no internationally agreed terminology.^3^ Clinicians identify complex physical and moral issues that occur when attempting to define what may constitute an act with the potential to hasten death (see Table 1). Acts that ‘hasten death’ are often seen as solely voluntary euthanasia and physician assisted dying.^4^ However, acts of care, which are relatively common in palliative care, such as withdrawal of life-sustaining treatment and the use of sedation may also be perceived as death hastening.^5^ For some people with a desire to hasten their death, the choice to voluntarily stop eating and drinking may be taken^6^ and can often be considered as a natural form of dying.^7^ Healthcare professionals may continue to have an active role in supporting patients who make this choice and not intervene in halting the dying process.^8^

**Table 1.** Definitions.

Term	Definition and intended aim	Intended purpose	Does this hasten death?
Euthanasia As used in Belgium, The Netherlands, Luxembourg and Columbia. Also permitted in jurisdictions in Australia when the patient is unable to administer the medication themselves, however this is classed under the term voluntary assisted dying.	Intentionally ending the life of a patient by a health care practitioner by means of active administration of a drug at that patient’s explicit request^3^	Healthcare – to support autonomous patient choice Patient death	Yes
Physician assisted suicide (PAS) Sometimes called assisted suicide – used in Switzerland, Luxembourg, Columbia Voluntary assisted dying – used in Australia Physician aid in dying or aid in dying – used in the USA Medical aid/assistance in dying (MAiD) – used in Canada	Intentionally ending the life of a patient by a health care practitioner by means of providing or prescribing drugs for a patient to use to end their own life^3^ Includes both euthanasia and physician assisted suicide, with both physicians and nurse practitioners being allowed to undertake the practices.^3^	Healthcare – to support autonomous patient choice Patient death	Yes
Sedation/palliative sedation	The relief of suffering through monitored proportionate use of medication intended to reduce consciousness in patients with life-limiting illness.^9–11^ An umbrella term referring to the management of refractory symptoms along a spectrum from continuous deep sedation though to ‘light’ sedation.^12^ The use of low doses of Midazolam or opioids for symptom management, where sedation is not the primary aim, is not considered palliative sedation within this context.^10^	Healthcare – to support autonomous patient choice. Reduction of consciousness to relieve suffering.^10^ Promote comfort at end of life.^12^ Patient – reduction of awareness or death^13,14^	Yes^10,12,15,16^ No^17–21^ Maybe^20,22^ Uncertainty as to its status. Death as an unintended consequence of the intervention.
Withdrawal of life-sustaining treatment	Withholding or withdrawing medical treatment from a person due to medical futility or a person’s voluntary and competent request.^23^ These may also be referred to as decisions to limit treatment.^24^ A range of interventions, including but not limited to, withdrawal or weaning of mechanical ventilation,^25–28^ oxygen^29^ or cardiac system support^28^ and discontinuing antibiotics,^28^ parental nutrition and hydration,^30–32^ blood transfusions,^33^ chemotherapy^34^ or haemodialsyis^35–39^	Healthcare professionals – Support patient choice in care and reduce treatment burden in the last days of life.^40^ Patient – reduce treatment burden but may also be to avoid life prolongation.	Dependent upon the intervention and clinical condition. Timescale to death is also variable dependent upon the intervention
Voluntary stopping eating and drinking	Where a person actively choses to stop eating and/or drinking with the intention to hasten death.^6^ A self-initiated, active and ongoing effort, by a person with mental capacity, to accelerate dying in the contexts of suffering refractory to aggressive disease and symptom management, which can occur irrespective of care setting.^6,41–44^ Voluntary stopping eating and drinking does not involve active intervention from healthcare professionals to withdraw treatment	Healthcare – to support patient autonomous choice Patient – to hasten death^44–46^	Yes^7,45–47^

The complexity in defining these care acts also links to their ethical status. Primarily whether death, in this context, is an intended or unintended consequence or side effect of the act for the healthcare professional^5,48–56^ Although it is recognised within legal and ethical frameworks where these differ from assisted death,^57^ it can be difficult to delineate the intentions of patients and healthcare professionals within existing frameworks.^15^ There may also be a disconnect between doctors’ and nurses’ perceptions of acts that hold the potential to hasten death,^58–65^ with nurses more likely to experience internal conflict and increased uncertainty about the nature of these interventions.^15,22,62^ This uncertainty is more marked within the use of sedation than other interventions.^9,66^ Whilst attempts are made within the literature to decrease the ambiguity that nurses feel about whether care may hasten death, it is also recognised that this originates primarily from physician authored papers. The focus on ‘educating’ nurses to help them gain moral clarity may serve to further marginalise their concerns about these acts.^67^

The term ‘experience’ can often be used without explanation as to what it may refer to.^68^ Experience can be considered as an active ‘intersubjective, social and political’ process that people enter through in order to create meaning.^69^ A relational understanding and expectations of the nurse-patient relationship defines the nurse’s experience, yet their experiences are commonly amalgamated with other healthcare professionals, most specifically physicians. Nurses identify their role as unique within the healthcare team. The frequency and intimacy of patient contact, seeing themselves as a patient advocate, and coordinating care to support patients to navigate the healthcare system are all cited as specifically defining their role in palliative care.^70,71^ The dynamics with the wider healthcare team are also considered as potentially impacting how care delivery is experienced.^70,71^ Both nurses and physicians feel that nurses are well placed to identify suffering and establish if symptoms are poorly managed or whether further treatment may be futile.^22,72^ However, the level of involvement nurses have in decision-making is noted as an important factor that impacts upon the quality of their experience delivering care; often primarily influenced by the physician.^72^ Nurses being required to undertake care they perceive not in the patient’s best interest or where they feel they are witnessing unnecessary and iatrogenic suffering can result in moral injury.^73^ While there appears to be some issues in common for all healthcare professionals, synthesising research that solely considers nurses will allow a greater understanding of their own nuanced experiences.

This review aims to synthesise evidence across acts that nurses may perceive as death hastening to yield new knowledge of the experience of providing care that may or will hasten death. Understanding where, or if, comparable experiences exist could help develop knowledge related to the practical, ethical and moral complexities nurses report when providing care at the end of life. Finally, this review aims to amplify the nursing voice within this complex and often divisive topic.

### Review question

What can be learnt from synthesising qualitative research findings on the lived experiences of nurses when involved with acts that may be perceived as death hastening?

### Methodology/methods

A qualitative evidence synthesis was undertaken utilising thematic synthesis, as described by Thomas and Harden.^74^ The research question considers experience, as such a focus on primary qualitative research, which is supported by this approach, is most appropriate. Thematic synthesis is valuable when the research question aims to gain a deeper understanding of an unknown issue, rather than theory generation. Thomas and Harden^74^ state that, within this form of synthesis, it may not be possible, or required, to locate all available evidence. However, the strategy focusses on ensuring that no new research would alter the ‘conceptual synthesis’.^74^ Reverse and forward citation checking and reviewing recent documents published in the author’s country were included in the search strategy. This process stopped when there was agreement across authors that the results were conceptually rich. Analysis and synthesis were undertaken using the three-step approach.^74^

1. Coding text
2. Developing descriptive themes
3. Generating analytical themes.

The review exists within a social constructivist paradigm, where individuals’ experiences of the world shape understanding and meaning-making.^75^ This recognises the overt importance of the social context of the research and the influence of positionality within the analysis. This evidence synthesis is reported in line with the enhanced transparency in reporting the synthesis of qualitative research (ENTREQ) checklist.^76^

### Search strategy

The search strategy and subsequent inclusion/exclusion criteria were developed using SPIDER.^77^ SPIDER was utilised to facilitate a search that considers experience across interventions. To increase the sensitivity of the search strategy, the research design was not specified within the search terms. The lead author (VA) ran initial searches in December 2022 and updated in August 2024 using the Cumulative Index to Nursing and Allied Health Literature (CINAHL), PsycINFO, and MEDLINE databases. The search strategy was developed alongside a Lancaster University subject specialist librarian using Medical Subject Heading (MeSH) terms and database specific linked suggested terms and was tested against two known papers. The search terms are identified in Table 2 and results were obtained by linking searches with the Boolean operator AND.

**Table 2.** Search terms.

Database	Phenomenon of interest	Sample
CINAHL	TI ( (MH ‘Suicide, Assisted’) OR (MH ‘Euthanasia, Passive’) OR (MH ‘Euthanasia+’) OR ((stop* OR cease OR withdraw*) N5 (food OR drink* OR sustan* OR treat*)) OR (euthan*) OR (assisted-dying) OR (assisted-suicide) OR (suicide) OR (palli* N5 seda*) OR ((assist* OR haste*) N5 (death OR dying OR die)) ) OR AB ( (MH ‘Suicide, Assisted’) OR (MH ‘Euthanasia, Passive’) OR (MH ‘Euthanasia+’) OR ((stop* OR cease OR withdraw*) N5 (food OR drink* OR sustan* OR treat*)) OR (euthan*) OR (assisted-dying) OR (assisted-suicide) OR (suicide) OR (palli* N5 seda*) OR ((assist* OR haste*) N5 (death OR dying OR die)) ) Limiters 01/01/1997 Human	(MH ‘Nurses’) OR Nurs*
PsychINFO	TI ( (DE ‘Euthanasia’) OR (DE ‘Assisted Suicide’) OR ((stop* OR cease OR withdraw*) N5 (food OR drink* OR sustan* OR treat*)) OR (euthan*) OR (assisted-dying) OR (assisted-suicide) OR (suicide) OR (palli* N5 seda*) OR ((assist* OR haste*) N5 (death OR dying OR die)) ) OR AB ( (DE ‘Euthanasia’) OR (DE ‘Assisted Suicide’) OR ((stop* OR cease OR withdraw*) N5 (food OR drink* OR sustan* OR treat*)) OR (euthan*) OR (assisted-dying) OR (assisted-suicide) OR (suicide) OR (palli* N5 seda*) OR ((assist* OR haste*) N5 (death OR dying OR die)) ) Limiters 01/01/1997 Human	TI ( DE ‘Nurses’ OR DE ‘Nursing’ OR Nurs* ) OR AB ( DE ‘Nurses’ OR DE ‘Nursing’ OR Nurs* )
MEDLINE	AB ( (MH ‘Euthanasia’) OR (MH ‘Euthanasia, Active, Voluntary’) OR (MH ‘Suicide, Assisted’) OR (MH ‘Euthanasia, Active’) OR (MH ‘Euthanasia, Passive’) OR (MH ‘Right to Die’) OR ((stop* OR cease OR withdraw*) N5 (food OR drink* OR sustan* OR treat*)) OR (euthan*) OR (assisted-dying) OR (assisted-suicide) OR (suicide) OR (palli* N5 seda*) OR ((assist* OR haste*) N5 (death OR dying OR die)) ) OR TI ( (MH ‘Euthanasia’) OR (MH ‘Euthanasia, Active, Voluntary’) OR (MH ‘Suicide, Assisted’) OR (MH ‘Euthanasia, Active’) OR (MH ‘Euthanasia, Passive’) OR (MH ‘Right to Die’) OR ((stop* OR cease OR withdraw*) N5 (food OR drink* OR sustan* OR treat*)) OR (euthan*) OR (assisted-dying) OR (assisted-suicide) OR (suicide) OR (palli* N5 seda*) OR ((assist* OR haste*) N5 (death OR dying OR die)) ) Limiters 01/01/1997 Human	AB ( (MH ‘Nurses’) OR Nurs* ) OR TI ( (MH ‘Nurses’) OR Nurs*

A manual search was undertaken through citation checking of reference lists and forward tracking of citations within included studies to ensure no papers had been omitted. A selection of reference lists of legalisation and policy documents local to the author were also reviewed, although this process did not add any further papers.

### Inclusion/exclusion criteria

The inclusion/exclusion criteria are given in Table 3.

**Table 3.** Inclusion/exclusion criteria.

Criterion	Inclusion	Exclusion
Population	Registered Nurses Patients over the age of 18 years	Registered nursing associates Studies with multiple healthcare professionals in the sample Patients or informal carers Volunteers
Intervention	Assisted dying/assisted suicide/euthanasia Withdrawal of life-sustaining treatment Voluntary stopping eating or drinking Sedation at end of life	Requests for assistance to die due to a mental health diagnosis Non-voluntary euthanasia Veterinarian studies
Outcome	Experience within the provision of care	Studies solely relating to the description of the medical intervention and process of assisted dying Attitudes/opinions to assistance to dieExperience of provision of care – considered solely in terms of level of involvement
Language	Findings published or available in the English language	
Dates	Research undertaken after 1997, in line with first legalisation of assisted dying.	Timeline (relating to legality of assisted dying) is not able to be established within the paper.
Study design	Qualitative	Quantitative Mixed methodology Literature or systematic reviews. Grey literature including blogs Opinion pieces, editorials and commentaries Book/book chapters

The papers’ titles were initially evaluated against the inclusion criteria. Abstracts and full text papers were read if they appeared to meet the inclusion criteria following the title search. All included papers were then re-read to ensure their appropriate inclusion. A second reviewer (JM) blind-reviewed 10% of titles (360 out of 3600 papers) from the initial search against the inclusion criteria. The process was undertaken utilising Rayann^®^ and the decisions were blinded until both reviewers had completed the evaluation process. Decision-making relating to the application of the inclusion criteria was discussed and recommendations were made to provide rigour to the process. An example of this was how the concept of experience was understood by both reviewers as this was central to the review process. There was consensus on which papers should be included.

### Data extraction, appraisal and synthesis

Data were extracted using a tool developed from the work of Noyes,^78^ supporting the extraction of large amounts of narrative data verbatim from the research. The tool was modified to include data relating to the review question, including the legality of assisted dying. The second reviewer (JM) undertook data extraction on 10% of the included papers (3 of 23) to clarity check the tool and protocol for appropriate data extraction. Uncertainty was resolved through initial discussion and changes made to the extraction tool to capture necessary data. The studies were quality assessed alongside data extraction^79^ using the Joanna Briggs Institute quality appraisal tool.^80^ No papers were excluded based upon this assessment, although data quality was considered reflexively during the synthesis, in keeping with the method.^74^

Coding and theme development were undertaken inductively. Analytical themes were developed following the identification of cross cutting, comparative and parallel descriptive themes.^81^ A hybrid approach to coding was undertaken using both Nvivo^®^ for descriptive themes and moving to pen/paper for the development of analytical themes. Reflexivity is fundamental in the development of a rigorous thematic synthesis and therefore regular discussions and reflections with the supervisory team (NP and LM) were undertaken.

## Findings

**Figure 1.**
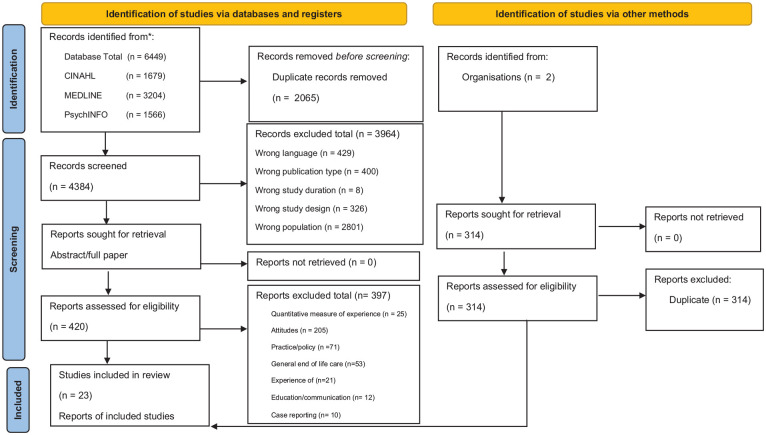
PRISMA.

From 4384 papers assessed for eligibility, a total of 23 papers were identified relating to nurses’ experience of acts that may potentially hasten dying (see PRISMA Figure 1). Thirteen relate to assisted dying including six from Canada, five from Belgium and two from the USA. These papers are from areas with permissive assisted dying legislature with the exception of De Bal et al.,^94^ Dierckx de Casterle et al.,^95^ Schwarz^103^ and Volker.^104^ Three papers consider sedation, these are from Canada, the UK and the Netherlands and seven consider withdrawal of life-sustaining treatment all in jurisdictions without permissive assisted dying legislature at the time of the study. All papers relating to withdrawal of life-sustaining treatment were based in intensive care settings. No papers considering voluntary stopping eating and drinking met the inclusion criteria. Table 4 provides a summary of included papers.

**Table 4.** Summary of included papers.

Author and year	Country	Research question/s or aims	Sample	Data collection	Methodology and analysis	Key findings
Palliative sedation						
Beel et al.^82^	Canada: Manitoba Status of assisted dying: Not legal (prior to legislature)	Explore nurses’ knowledge, attitudes and the meaning nurses attributed to the use of palliative sedation in dying adult patients in a palliative care unit.	Purposive sampling. 10 nurses within specialist palliative care unit, experience of palliative sedation.	Individual semi-structured face to face interviews.	Qualitative study using symbolic interactionalism. Thematic content analysis.	Theme Working your way through the quagmire. Sub themes Definitional quagmire – Difficulty in the definition of palliative sedation. Indications for use quagmire – Uncertainty of when to use palliative sedation. The need to create comfort. The traumatic effect of not managing symptoms. Team and family readiness for administration.
De Vries and Plaskota^83^	United Kingdom Status of assisted dying: Not legal	The experiences of hospice nurses when administering palliative sedation in an attempt to manage the terminal restlessness experienced by cancer patients.	Purposive sampling. Seven nurses within hospice, with experience of palliative sedation within the past year.	Individual semi-structured interviews.	Qualitative study using a Phenomenological approach.	Theme Facilitating a peaceful death Sub-themes Decision making and ethical and emotional conflict .Causing the death .Sedating young people .Requests for sedation and believing that hospice was a place where death is hastened .Being supported.
Lokker et al.^84^	The Netherlands Status of assisted dying: Legal	Explore nurses’ reports on the practice of palliative sedation focussing on their experiences with pressure, dilemmas and morally distressing situations.	Convenience sampling. 36 nurses across a range of clinical area recruited through involvement in a previous study.	Individual semi-structured interviews.	No description of methodology. Analysis undertaken using constant comparative method.	Themes Experiencing constraints preventing action. Experiencing pressure to act .Subthemes Experiencing pressure to act before and during the palliative sedation process.
Withdrawal of life-sustaining treatment						
Efstathiou and Walker^85^	United Kingdom Status of assisted dying: Not legal	Explore the experiences of intensive care nurses who provided end-of-life care to adult patients and their families after a decision had been taken to withdraw treatment.	Purposive sampling. 13 nurses working in intensive care from one hospital.	Individual semi-structured interviews.	Descriptive exploratory qualitative approach. Data analysed using interpretative phenomenological analysis.	Themes Caring for the dying patient and their family .Providing and encouraging presence. Reconnecting the patient and their family .Dealing with emotions and ambiguity.
Halcomb et al.^86^	Australia Status of assisted dying: Not legal (prior to legislature)	Investigate the experience of nurses caring for clients in the ICU having treatment withdrawn or withheld.	Convenience sampling. 10 nurses with experience of withdrawal of life-sustaining treatment.	Individual conversational interview.	Qualitative study using Phenomenological approach.	Themes Comfort and care .Tension and conflict .Do no harm. Nurse-family relationships .Invisibility of grief and suffering.
Hov et al.^87^	Norway Status of assisted dying: Legal	Acquire a deeper understanding of what it is to be an intensive care nurse in situations related to questions of withholding or withdrawing curative treatment.	Purposive sampling. A total of 14 (female) nurses working in intensive therapy unit.	Two focussed non-structured group interviews.	Qualitative study using interpretive phenomenology.	Themes Loneliness in responsibility. Alternation between optimism and pessimism. Uncertainty – a constant shadow. Professional pride despite little formal influence.
Johnson and Jack^88^	United Kingdom Status of assisted dying: Not legal	Explore experiences of high dependency unit (HDU) nurses caring for patients approaching withdrawal of life-sustaining treatment. Highlight any support or needs they may have.	Purposive sampling. 15 nurses with greater than 12 months experience working within an HDU	Individual semi-structured interviews	Qualitative descriptive methodology	Theme Supporting HDU nurses to provide and survive withdrawal of life-sustaining treatment .Sub-themes Conflict in decision making .Prolonging distress .Moral distress .The need to talk .The need for further education.
McMillen^89^	United Kingdom Status of assisted dying: Not legal	Explore the perceptions, feelings and experiences of nurses relating to end of life decision making in one ICU in the United Kingdom.	Purposive sampling. 8 nurses working in the same ICU.	Individual semi-structured interviews.	Constructivist grounded theory. Analysis using framework analysis.	Theme The nurses role .Sub themes Experience counts. Not really a nurses decision .Planting the seed. Supporting the family .Being a patient advocate .Theme Perceptions of the withdrawal of treatment. Sub themes Getting the timing right.Emotional labour.
Taylor et al.^90^	Norway Status of assisted dying: Not legal	Explore the experience of intensive care nurses when participating in the withdrawal of life-sustaining treatments from intensive care unit patients.	Purposive sampling. 9 nurses with greater than 2 years’ experience and experience of withdrawal of life-sustaining treatment.	Individual semi-structured interviews.	Qualitative descriptive and exploratory design.	Categories ICU nurses’ experiences of stress in the process of treatment withdrawal .A requirement for interdisciplinary support and cooperation .Elements to achieve a dignified treatment withdrawal process.
Verderspank – Wright et al.^91^	Canada Status of assisted dying: Not legal (prior to legislature)	Explore the experiences of critical care nurses caring for patients through withdrawal of life-sustaining treatment. Identify factors which nurses feel hinder or facilitate them caring for these patients.	Purposive sampling. 6 nurses with over 6 months experience and cared for someone during withdrawal of life-sustaining treatment.	Individual semi-structured interviews.	Qualitative phenomenological study.	Overarching concept: Trying to do the right thing. Themes A journey: creating comfort along the way. Working in professional angst .Providing memories.
Assisted dying						
Bellens et al.^92^	Belgium Status of assisted dying: Legal	To explore how Flemish nurses working in hospitals and home care experience their involvement in the care of patients requesting euthanasia 15 years after the legalisation of euthanasia.	Purposive and snowball sampling. 26 nurses working in hospital or home care with experience of euthanasia	Individual semi-structured interviews.	Qualitative study using grounded theory Analysis informed by Qualitative Analysis Guide of Leuven.	Themes Intense and not unambiguous .Professional fulfilment .Frustration.
Beuthin et al.^93^	Canada – British Columbia Status of assisted dying: Legal	Understand the range of nurses’ experience in providing care for someone choosing MAiD, whether directly aiding, providing supportive care or declining to participate.	Purposive sampling. 17 nurses working across varied clinical setting.	Individual semi-structured interviews either face to face or via telephone.	Qualitative design using narrative enquiry. Data analysis using thematic analysis.	Theme Profession of nursing .Subthemes Holistic care without judgment .Advocating choice .Supporting a good death .Theme Personal impact .Subthemes Being pioneers .Sensemaking: Taking a stand. Experiencing emotional spectrum .Theme Nursing practice.
De Bal et al.^94^	Belgium – Flanders Status of assisted dying: Not legal (prior to legislature)	Explore nurses’ involvement in the care for patients requesting euthanasia.	Purposive sampling. 15 nurses working in two acute hospitals in Flanders.	Individual semi-structured interviews.	Qualitative study using grounded theory. Constant comparison method, in line with grounded theory approach.	Themes The nurses’ conflicted feelings about (their involvement in) euthanasia .Powerless: the central emotion experienced by participants .The context of nursing care .Nurses’ key role in caring for patients with a euthanasia request: the process model.
Dierckx de Casterle et al.^95^	Belgium – Flanders Status of assisted dying: Not legal (prior to legislature)	Palliative care nurses’ views on their involvement in the care process surrounding euthanasia.	Purposive sampling. 12 nurses working in a palliative care setting.	Individual semi-structured interviews.	Qualitative study using grounded theory. Constant comparison method in line with grounded theory approach.	Themes Hearing a request for euthanasia .Participation in decision-making process. Participation in the execution of euthanasia .Supporting family members and colleagues.
Denier et al.^96^	Belgium – Flanders Status of assisted dying: Legal	What does participation in the euthanasia case process actually mean for the nurse involved?	Purposive and theoretical sampling. 18 nurses working in an acute hospital setting.	Individual semi-structured interviews – recalling a recent case of a euthanasia request.	Qualitative study using grounded theory approach .Interview transcripts systematically examined to identify themes.	Nurses had a procedural, action-focussed perspective or an existential-interpretative perspective which determined their view on the process. This manifests during the process, understanding of the purpose of involvement, extent of involvement and how it may evolve over time.
Denier et al.^97^	Belgium – Flanders Status of assisted dying: Legal	To explore nurses’ experiences in caring for patients requesting euthanasia.	Purposive and theoretical sampling. 18 nurses working in an acute hospital setting.	Individual semi-structured interviews, paper using the same dataset as Denier et al.^97^	Qualitative study using grounded theory approach Analysis method not described.	Themes Intense .Experiences which changed and developed over time .Various factors which positively or negatively influenced the nurses’ experience of the euthanasia care process.
Hébert and Asri^98^	Canada – Quebec Status of assisted dying: Legal	Explore how Quebec nurses personally and professionally face the new practice of MAiD and their role evolution. To describe the paradoxes experienced by nurses	Theoretical sampling. 37 nurses within who had participated in MAiD. Nurses were French speaking no discussion about translation	Individual semi-structured interviews and focus groups	Qualitative study using grounded theory Constant comparison method in line with grounded theory approach	Nurses experienced a wide range of paradoxes during MAiD centred around eight elements Confrontation about death Choice Time of death Emotional load New Bill Relationships with the person Communication skills Healthcare settings
Pesut et al.^99^	Canada Status of assisted dying: Legal	Understand the implications of a legislated approach to assisted death for nurses’ experiences and nursing practice.	Convenience, purposive and snowball sampling. 59 nurses who had been involved or conscientiously object to MAiD.	Individual semi-structured interviews.	Qualitative study, interpretative description. Constant comparison method.	Themes The leadership taken by influential people within systems .The presence and nature of a multi-disciplinary team. The systems’ complexity and capacity to support MAiD.
Pesut et al.^100^	Canada Status of assisted dying: Legal	To describe nurses’ moral experiences of MAiD in the Canadian context.	Convenience, purposive and snowball sampling. 59 nurses who had been involved or conscientiously object to MAiD.	Individual semi-structured interviews. Uses the same data set as Pesut et al.^99^	Qualitative study, interpretative description. Constant comparative method.	Theme Willingness to participate in MAiD: Morally relevant factors .Sub themes Family and community influence\ Professional experiences. Proximity to the act of MAiD. Theme Experience of MAiD. Sub themes Emotional experiences. Attributions. Theme Moral waypoints .Sub themes Patient choice, control and certainty. It’s not about me. Nurses’ role in alleviating suffering. Moral consistency. Reflections on the afterlife. Peace and gratitude.
Pesut et al.^101^	Canada – British Columbia Status of assisted dying: Legal	Describe the experiences of nurses and nurse practitioners with the implementation and ongoing development of MAID from Bill C-14 to Bill C-7.	Purposive and snowball sampling. 50 nurses working across any setting within English-speaking provinces.	Individual semi-structured interviews via telephone.	Qualitative longitudinal descriptive study Constant comparative analysis.	Theme Implementing Bill C-14: transitions and challenges. Sub themes Normalised to a point. From secrecy to visibility. Greater accessibility. Trusting the process .Increase case, complexity and workload. Remuneration challenges. MAiD and palliative care: tensions and synergies. Patient choice and inequities in access. Benefits and challenges of programme integration .Theme Eligibility and safeguards under C-14 .Sub themes Evolving gestalt of eligibility .Stress of telling someone they are ineligible .Finding a wat to make someone eligible .Waiting periods and final consent. Theme Anticipating Bill C-7 .Sub themes New population brings new complexities .A cry for help not MAiD.
Pesut et al.^102^	Canada	To explore the evolving practices related to MAID in Canada from the perspective of nurses.	Convenience, purposive and snowball sampling. 35 nurses with clinical experience of MAiD.	Individual semi-structured interviews.	Interpretive descriptive study. Constant comparative analysis.	Theme Introducing MAID as part of Advance Care Planning .Living beyond capacity: waivers of consent. Hastened death when death is not foreseeable: many shades of grey.
Schwarz^103^	United States of America Status of assisted dying: Not legal	What is the nature of the experience of being asked to help someone die?	Voluntary response sample. 10 nurses who believed a competent person had asked them for help dying.	Individual interviews.	Van-Manen’s phenomenology and thematic summary.	Theme Being open to hear and hearing Interpreting and responding to the meaning. Sub themes Multiple meanings of hastening death. Use of double-effect reasoning and the meaning of intentions. Theme Responding to persistent requests for AID. Sub themes Finding a moral line .Conflicts and control over dying. Providing direct AID.
Volker^104^	United States of America Inc. Oregon Status of assisted dying: Not legal	To explore oncology nurses’ experience with receiving requests for assisted dying from terminally ill patients with cancer.	Purposive sampling. 40 Clinical Nurse Specialists in oncology.	Submitted written stories, 48 included.	Descriptive, naturalistic study. Thematic analysis interpretive interactionism.	Theme Control .Subthemes Cry for help .Hastening the process. What if. . . . . Managing the morphine .Countering with palliative care. Theme Conflict .Subtheme Collision of values. Distress .Theme Covert communication .Subtheme The dialogue around the request .The silent knowing. Theme The enduring influence. Sub themes The unforgettable. Lessons learnt.

Within this synthesis, the term ‘other acts that may hasten death’ is used to represent withdrawal of life-sustaining treatment and sedation. The term ‘assisted dying’ has been chosen to encompass interventions across physician assisted suicide, euthanasia and MAiD. This is used as a neutral, umbrella term for acts undertaken with the active intention of inducing death, in keeping with suggested international convention.^3,105,106^

An overarching theme, *the emotional labour of care* was developed with three subthemes that influence the experience of delivering care and subsequent levels emotional labour involved. The sub themes are (1) experiencing personal and professional conflicts, (2) the provision of ‘normal(ised)’ care and (3) perceptions of palliative care as a proxy for hastening death. Table 5 identifies how the papers are represented within the themes.

**Table 5.** Themes.

Theme	Assisted dying	Other acts that may hasten death
Overarching theme: The emotional labour of care	Showing and regulating emotions^92–101^	Showing and regulating emotions^83,85–90^
	Positive emotions^92–94,97,98,100,102^	Positive emotions^82,86^
	Negative emotions^92–94,97–100,102^	Negative emotions^82,84,87,86,88–90^
	The impact of witnessing suffering^82,84,86–88,93,98–100^	The impact of witnessing suffering^82,84,86–88^
Experiencing personal and professional conflicts	Moral tension^93–95,97,102,103^	Moral tension^86,90^
	The nurse’s role in decision-making^94,95,97,102^	The nurse’s role in decision-making^82,83,84,86–91^
	Working within boundaries^92,94–96,98–100,102^	Working within boundaries^82,83,85–87,91^
The provision of ‘normal(ised)’ care	Controlling the point of death^92,93,95,97,99,100,103^	Controlling the point of death^82–86,89–91^
	The nurse’s perception of the dying process^92,93,95,97–100,103,104^	The nurse’s perception of the dying process^82–84,86,88–91^
Perceptions of palliative care as a proxy for hastening death	Family perceptions^94,97,104^	Family perceptions^83,84,87,88^
	The nurse’s uncertainty^93,94,103,104^	The nurse’s uncertainty^82,83,87,88^
	Navigating with and through language^94,97,100–104^	Navigating with and through language^82,83,84,88,89^

### The emotional labour of care

The emotional labour associated with nurses’ involvement in care that may hasten death is an overarching theme within the studies. Emotional labour is defined as ‘the management of feeling to create a publicly observable facial and bodily display’ (p. 7)^107^. It is an active process where behaviours develop in line with social expectations of professional roles. Emotions can be suppressed and outwardly expressed emotions and behaviours can be performative depending upon situational expectations,^107^ which is seen consistently within the studies. Overall, nurses identify a requirement to understand, navigate and conform to expected behaviours, when involved with all acts that may hasten death, which requires emotional labour. Opinion varies amongst nurses in the studies about what behaviours may be appropriate, including showing emotions during care delivery. When involved in the withdrawal of life-sustaining treatment, nurses feel showing emotions publicly can be appropriate^85^ although they also described needing to hide these from the wider team.^86,87^ When caring for people having an assisted death, any outward physical emotional response is generally removed from any public view.^94,96,97^ Within palliative sedation, nurses frequently describe situations they find challenging linked to witnessing suffering, though the expression of emotion is not considered.

Across interventions, nurses report involvement in care impacting both positively^82,86,92–94,97,98,100,102^ and negatively^82,84,87–90,92–94,97–100,102^ on them. Experiences perceived as negative are reported as impacting nurses’ personal lives with examples of them ‘taking it home with you’.^86,88–90,94,98,100^ Within all acts, nurses discuss emotional detachment as a useful strategy to manage emotional labour,^86,87,90,95,96,98^ although some do not routinely use this in practice.^94,100^

As professionals nurses’ sympathy extended to their patients, but a certain emotional distance was maintained. Many nurses underlined the importance of psychologically releasing the patient’s request. Not all nurses succeeded in maintaining this emotional distance. . . . . .Non-palliative care nurses in particular took their experiences home after their shift^94^ p 596

Instead, nurses commonly feel required to regulate negative emotions while providing patient care in order to manage their own well-being.^87,88,94–97,100^ Across all acts, emotional labour is reported to diminish as nurses gain experience with providing care, both through desensitisation to witnessing dying and through the development of clinical skills.^83–85,89,92,96–98,100,101^

The intensity and level of emotional labour required are consistently considered by nurses as linked to a desire to help when witnessing suffering. Nurses specifically report that iatrogenic suffering increases the emotional labour required to provide compassionate care^82,84,86–88,93,98–100^ and was also considered as motivation for involvement within assisted dying.^93,98,100^

That first experience troubled me. Not because of the experience itself, but because of all the previous events that could have ended much better if MAID had been available before. I have images of people I have accompanied in painful end-of-life situations (P17). We do MAID, it is not painful. There is pain, but there is much less pain and suffering compared to someone slowly dying for two or 3 days with pulmonary complications (P31).^98^ p. 1638

Nurses who decline to be involved in assisted dying due to moral objection (conscientious objection) also report aspects of care that generate emotional labour. The requirement to deliver care for a patient knowing they wish to have an assisted death or providing care for other patients when an assisted death is occurring are reported as burdensome.^93,100^ The position as ‘conscientious objector’ also makes them feel vulnerable, with support depending upon individual manager’s stance rather than organisational policy.^93,99^ The lack of organisational support leads nurses to feel they must self-regulate emotional responses due to the uncertainty of the reactions of team members. As such, active involvement in assisting dying cannot be considered as the sole cause of emotional labour in these cases.

### Experiencing personal and professional conflicts

This theme can be summarised as elements that cause personal and professional conflict, which affects the levels of emotional labour required to provide patient care. Conflict occurs when nurses, to meet professional expectations, are required to work beyond perceived professional or moral boundaries. The professional obligations nurses feel towards patients mean they will offer support irrespective of moral tensions that may occur.^86,90,93–95,97,102,103^ This was consistently observed across all acts that may hasten death.

Conflicts experienced by nurses are, most commonly, linked to interprofessional tension and are a significant consideration in all studies. Most notable for nurses is the sense that their input is not valued and their role in clinical decision-making is not recognised within the healthcare system.^82,84,86–90,94,97^ Nurses describe themselves, within the healthcare team, as closest to patients and therefore best placed to accurately represent the patient’s wishes and clinical condition to others.^84,86–88,94,95,97,102^ There was agreement, within other acts that may hasten death, that nurses will often find themeslves alone in navigating the consequences of these decisions with patients and families.^83,84,86,87,90,91,94^ The quote from the Australian paper illustrates the consequent challenge for nurses to (re)present decisions that they may disagree with and deliver care they feel not to be in the patient’s best interest. Essentially becoming the representative of the plan of care on behalf of the healthcare systems.

When the doctors sought to withdraw/withhold treatment, the participants expressed that they were often dissatisfied with the management of the situation. The most clearly articulated complaint was that whilst the doctors excluded nurses from the decision making and formulation of the management plan, once a decision is made to forego medical treatment the nurse is left to manage the dying process. ‘‘*The mind your business and I’ll make the decision, then they make their decision or have the family make the decision . . . and then leave you to deal with it. . . they do it all the time* . . . . . ’’.^86^ p. 218

To reduce emotional labour nurses need to feel supported by the clinical team and a shared philosophy of care is considered important.^82,83,86,92,94,95,98–100^. When mentioned, nurses feel that policy, no matter what that may be, is supportive as it legitimises the nurses’ actions and is perceived as constructing safe professional boundaries.^85,87,91,94,96,99^ Within other acts that may hasten death, a policy is more likely to be replaced by clinical guidelines causing ambiguity, with nurses describing feeling ‘caught’ between guidelines and individual physicians’ stance.^86,91,97^ Within assisted dying, tensions are reported as manifesting at an organisational level where nurses report a lack of input on structural and policy decisions.

Nurses, however sometimes found themselves trying to assist in a MAiD procedure with no practice guidelines in their places of work. This created uncertainty in their practice, particularly when nurses remained the primary caregivers of patients contemplating or undergoing MAiD^99^ p. 14

However, this was only observed when considering legislative changes through MAiD^100,102^ and may be representative of the seniority of the nurses involved in this study. Overall, the requirement to deliver care that juxtaposes their professional or personal viewpoint can leave nurses feeling disempowered, which adds to their emotional labour.

### The provision of ‘normal(ised)’ care

This theme reflects the perception that withdrawal of life-sustaining treatment and sedation are considered part of ‘normal’ healthcare, whereas assisted dying is perceived as something ‘other’. The term ‘normal(ised)’ is used to represent nurses’ perceptions that other acts that may hasten death, whilst not part of a ‘natural’ dying process, are accepted as part of ‘normal’ dying^86,88–90^. Assisted dying is considered as a significant event, and a non-standard, unnatural death,^92,93,95,97,98,100^ with nurses using terms such as ‘murder’ and ‘killing’ irrespective of their moral position.

*A reflective process was described by Donna: It was something really big for me when I saw the death certificate, it was this overwhelming feeling like, oh my gosh, I killed him. Because I think I truly believed that knowing his situation, and his. . . sort of isolation, that* had I not *been open to the conversation*, had I not *helped him access the information, that he probably would have never been able to access the MAiD services. . . . . . .*^93^ p. 516

Being the cause of death was not a significant narrative within other acts that may hasten death. McMillen^89^ is the only example where nurses consider whether their actions were the cause of the patient’s death.

*When asked if they played any part in the actual decision one participant replied*: ‘‘*No, and I don’t think I’d ever want to either because at the end of the day it’s somebody’s father, brother, mother whatever. No amount of money in the world could ever get me to make that decision I don’t think and I don’t know how they* (*the consultants*) *sleep at night sometimes*.’’ (*Nurse 3*)^89^ p. 254

Within this theme, there is a shared narrative about the point of death being controlled. Within other acts that may hasten death, this relates to healthcare-led interventions that nurses report undertaking to deliver compassionate care. Examples include delaying withdrawing life-sustaining treatment to wait for family members to be present and administration of sedation in response to perceptions of suffering.^82–86,89,103^ This control extends to nurses attempting to de-medicalise the experience of dying to optimise the experience for patients and those important to them. This links to the concept of an aesthetic death^90^ with examples including controlling the bedside environment, ensuring relatives have time with the person and titrating medication based upon the needs of those witnessing the dying process.^85,86,90,91^ Within assisted dying this manifested as nurses advocating for their patient’s choice and prioritising the patient within their caseload.^93,99,100^ For an assisted death, the point of death is invariably driven and controlled by the patient.^92,100,103^ This ‘othering’ of death within assisted dying is, perhaps, linked to control of the timing of death being situated outside of healthcare. A shift that is disruptive to the established or expected relationship between patients and healthcare professionals.

#### Perceptions of palliative care as a proxy for hastening death

Within this theme, the perception that palliative care provision can be seen as a proxy for hastening death is presented. In some cases, nurses perceive other acts that may hasten death as both physically and emotionally commensurate with an assisted death, and express this through implicit and explicit communication.^83,84,88,89,94,97,100,103,104^ For example, Denier et al.^97^ describe how some nurses consider sedation and assisted dying on a spectrum of interventions. Although not directly expressed, this is suggested through nurses’ concern about their own practices, such as ensuring sedation is used judiciously and anxiety if death is prolonged.^82,103,104^ Nurses use phrases such as ‘active dying’ and ‘very, very terminal agitation’ as a justification to use sedation for intractable symptoms^83^ or offering sedation as an alternative choice when assisted dying is requested.^94^

Nurses also feel required to navigate the consideration that the public may view palliative care as offering a means to hasten death.^87,88,97,104^ In cases of sedation, nurses report family members consider that hospices and palliative care teams become involved with the direct intention to shorten life.^83,84^ Nurses report shock when faced with requests to hasten death^83,108^ and feel responsible for addressing what they see as an ill-informed view. They describe wanting to be clear that palliative care interventions will not hasten death.^94^ However, the uncertainty some nurses express about the nature of these interventions, means this response can be incongruent with their personal view.

All of the nurses expressed uncertainty that palliative sedation could or would lead to the death of the patient and they repeatably reflected on this possibility. They all reported experiencing anxiety at some time about such an outcome, but all maintained the position of wanting what was deemed “best for the patient” at that time.^83^ p. 152

These responses, therefore, may be a performative action that nurses feel required to undertake as part of the ‘nursing role’.

Nurses identify difficulty in establishing an ‘acceptable’ moral line that connects their external position with their internal stance,^87,88,93,94,103^ which requires significant emotional labour. Nurses suggest a hesitancy to openly state the opinion that death may be hastened through the actions of healthcare professionals.

There seems to be a discrepancy between the criterion on life expectancy in the guideline and nurses’ views, where nurses appear to prefer a more limited life expectancy than the guideline. This may be related to nurses’ concerns that sedation might hasten death^84^ p. 160

As such, nurses may use innuendo and metaphor to help them navigate this ‘acceptable line’ with others, speaking to a shared implicit understanding around these interventions.^82,94,103,104^ In some cases, in countries where assisted dying was illegal, nurses used innuendo to give covert advice relating to medication use where a desire to die had been expressed.^104^

While the term “overdose” was never used, it still was something that was understood as an “option” that the patient would have if they felt it was necessary^104^ p. 45

Open discussions about the intention to hasten death are only reported in the assisted dying literature and legalisation of assisted dying is reported as facilitating more open discussions about care and reducing feelings of powerlessness for nurses.^94,97,101,102^ Overall, this theme reflects the complexity that nurses report navigating care that may hasten death and how nurses feel compelled, despite uncertainty, to present a narrative that interventions undertaken as part of routine palliative care do not hasten death.

## Discussion

### Main findings

Synthesis across acts of care that may, or intends to, hasten death has highlighted new parallels in experience for nurses. The emotional labour required to provide care is intensified by tensions created by nurses’ uncertainty as to whether the care they deliver may hasten death. Perhaps most significant is the recognition that supporting intentionally hastened death does not create distinctive challenges for nurses when compared to acts often provided within established palliative care. Actually, the synthesis appears to suggest the reverse. The normalisation of dying with medical intervention normalises the high levels of emotional labour needed to provide care. This challenges a narrative that, for nurses, involvement in assisted dying may feel distinct from involvement in established palliative care practices.

### What this study adds?

The findings within this synthesis build upon earlier reviews, which consider these interventions individually. These reviews describe the emotional impact on nurses, including tensions trying to navigate family and doctor interactions^71^ and the need to regulate emotions to deliver care.^19^ This review adds to the wide and often contradictory findings reported when healthcare professionals are overtly asked about attitudes towards, and experiences within, assisted dying.^109–112^

To address the review question, the data is understood through the lens of ethics of care. Care at the end of life is embedded with ethical and moral entanglements, and within the review, nurses consistently report ethical tensions linked to existent systemic powers. Ethics of care allows consideration of moral agency as embedded within interpersonal relationships. As such, decision-making is relationally oriented, directed towards care provision for others and understood through the interdependencies of relational responsibility bound within institutions.^113–115^ Nurses’ experience, in this context, is created through engagement with the life of the patient, the embodied physical and emotional spaces that nurses are placed within and who nurses spend time with during care delivery.^71,116–118^ Within the assisted dying literature, the predominately important relationship for nurses was between them and the patient and optimising the experience for the person dying.^97,101^ Whereas for other acts that may hasten death, nurses consider those with the patient as most important and place focus on optimising their experience of dying.^85,86,90^ This difference is perhaps influenced by the ability of the patient to engage with care decisions. Within other acts that may hasten death, patients are more likely to be semi-comatosed or in a medically induced coma and therefore conversations will be focussed upon those at the bedside. The intention for the nurses is to support families in creating a positive lasting impression of dying for those with the patient^119,120^ and linking to the provision of an ‘aesthetic’ death. However, this seemingly engenders the performative language and actions identified in the review, where the intention to communicate complex ethical decision-making is undertaken in ways to avoid misinterpretation.

The ‘spaciotemporal and bodily proximity’ of nurses with patients can make them uniquely placed to understand the patient’s needs, which nurses also reported within the review,^70,117^ yet ‘institutional space’ is needed to support their involvement in decision-making.^113^ ‘Institutional space’, in this context, refers to a philosophical space that supports nurses to utilise their skills and recognises the unique roles different healthcare professionals have in providing holistic care. Within assisted dying, nurses appear to be given ‘institutional space’, seeing themselves as taking a more proactive role in leading care,^117,121^ and as such feelings of disempowerment were not commonly reported. ‘Institutional space’ here provides a culture of support to actively advocate for patients, allowing nurses to influence the structures they worked within, reflective of their clinical experience.^122,123^ More commonly, nurses involved in acts that potentially hasten death reference a lack of agency in care and a culture of ‘getting on with it’^86^ adding to the emotional labour required. It is also important to recognise that the formal practical and emotional support for nurses reported as embedded within assisted dying services, are not reflected across other interventions that may hasten death. This is despite positive associations between self-compassion and the provision of compassionate care.^124^

Nurses ‘proximity’ can also increase the emotional labour involved providing in care.^125^ Witnessing suffering is perceived as an expected aspect of the nurse’s role, required to be managed silently.^126^ This links to a hegemonic and gendered expectation that nurses engage more in emotional labour in comparison to other healthcare professionals.^127^ The parallels within emotional labour noted in the review occur as nurses routinely place more value on the well-being of others than their own. Whilst the expectation to regulate emotions can also explain a commonality of moral and emotional dissonance identified within the review, which appears distinct to nursing literature. Doctors, for example, have been shown to often lack the social ‘permission’ to show emotion with colleagues when caring for critically unwell patients through the professional expectation of their role^128^ and describe experience centred through their own emotions rather than linked to patient experiences.^129^ As such, it is suggested that this form of emotional expression and subsequent emotional labour sits within a nursing space and feminised expectations of the nursing role.

The concept of normal(ised) care identified within this synthesis has significance for future developments in policy. Nurses do not currently see assisted dying as part of normal healthcare.^130^ It is significant to recognise that nurses consider controlling the dying process, through the care they deliver, as part of routine end-of-life care. However, when patients seek to control the point of their own death, nurses have difficulty seeing this as part of standard care. Using the term normal(ised) acknowledges that care at the end of life is often medicalised care influenced by factors within healthcare control. As such, no death considered in this review is considered natural or normal dying; it must only be considered normal within institutional healthcare.^131^ Nurses describe ownership to manipulate the care environment, undertaken in an attempt to (re)create a ‘natural’ or ‘normal’ death,^132^ which is valued and seen as part of compassionate nursing care.^118,133,134^ However, considering nurses’ feelings of disempowerment in decision-making,^135^ influencing the environment offers nurses a means of control and the knowledge of providing this aesthetic death is seen as an opportunity to reduce the emotional labour required to provide care.

When acts that may be considered as death hastening are subsumed into general nursing processes, the emotional labour to provide this care is not well recognised. Yet, this is incongruous with the subsequent emotional impact nurses report and is a significant tension this review highlights. As palliative care navigates through local, national and international paradigmatic shifts, due to increasing jurisdictions with assisted dying legislature, understanding the impact of delivering this care is vital. This review adds a nurse’s perspective to this discussion and emphasises the significant parallels in the experiences of nurses across care that may hasten death that may have previously been viewed as distinct.

## Strengths and limitations of the study

This review is the first synthesis considering the experiences of nurses across acts that intentionally and potentially hasten death. As such, this review serves to amplify nurses’ voices and, in some ways, attempts to address the subjugation of the value nurses bring to patient outcomes and experience, an aspect identified within the studies. The review takes a structured reflexive approach; therefore, offers one interpretation of the data. However, the use of a second reviewer throughout the iterative review process and the active engagement of the supervisory team adds rigour to the review findings.

The review highlights the paucity of evidence relating to nurse experience, which may also limit its transferability. There is a lack of research relating to the withdrawal of life-sustaining treatment outside of the intensive care unit and sedation outside of the hospice setting. Despite the inclusion of voluntary stopping eating and drinking, there was no evidence considering nurse’s experience in this area. A focus on these acts outside of ‘traditional’ settings would aid the development of a richer evidence base reflective of the places and people that deliver this care. This review must also be seen within the social context. The review can only present a Western-centric view of this topic, considering the geographical spread of the research, the diversity of participants and the role of nurses in these locations. This is a significant area for future development.

## Conclusion

Synthesising experience across acts that intentionally or potentially hasten death draws parallels between experiences previously viewed as distinct. Nurses are grappling with the complexities of understanding their roles and position with the wider team when providing care that may intentionally or potentially hasten death. Uncertainty relating to whether interventions may hasten death and a lack of agency within care delivery increases the emotional labour involved in providing care. The impact of iatrogenic suffering and the recognition that physical and emotional time is not dedicated to supporting nurses within normal(ised) care has significance for nurse wellbeing. From a nurse’s perspective, there may be more in common in the experience of providing care in these contexts than previously recognised.
